# Short-term effects of opioids during therapeutic hypothermia for neonatal encephalopathy

**DOI:** 10.3389/fped.2024.1405731

**Published:** 2024-11-13

**Authors:** Tina Jumani, Priya Mishra, Tonya Robinson, Jeffrey S. Shenberger, Jonathan M. Davis, Benjamin Sweigart, Rodica M. Turcu

**Affiliations:** ^1^ Department of Pediatrics, St. Elizabeth’s Medical Center, Brighton, MA, United States; ^2^ Department of Pediatrics, Thomas Jefferson University Hospital, Philadelphia, PA, United States; ^3^ Department of Pediatrics, Norton Children’s Hospital, Louisville, KY, United States; ^4^ Department of Pediatrics, Connecticut Children’s, Hartford, CT, United States; ^5^ Department of Pediatrics, Wake Forest Medical Center, Winston-Salem, NC, United States; ^6^ Department of Pediatrics, Tufts Medical Center, Boston, MA, United States; ^7^ Tufts Clinical and Translational Science Institute, Tufts Medical Center, Boston, MA, United States; ^8^ Department of Pediatrics, Massachusetts General Hospital, Boston, MA, United States

**Keywords:** neonatal encephalopathy, hypothermia, opioids, outcome, neurodevelopmental

## Abstract

**Objective:**

To examine the effects of opioids during therapeutic hypothermia (TH) on short-term outcomes in neonates with neonatal encephalopathy (NE).

**Methods:**

Multicenter retrospective study of neonates with moderate/severe NE from Jan. 2013–Feb 2021. Opioid exposure was classified as positive (>0.1 mg/kg) or negative (no exposure or ≤0.1 mg/kg) based on cumulative morphine milligram equivalents (MME). Negative binomial regression models were used to evaluate clinical outcomes.

**Results:**

One hundred and twenty neonates were included. Adjusted analyses indicated that opioid exposure was associated with an increase in (1) length of hospitalization, (2) hypotension/use of vasopressors, and (3) need for and longer duration of mechanical ventilation. Many findings persisted even after adjusting for site and the presence of confirmed seizures (a marker of disease severity).

**Discussion:**

Opioid use during TH was associated with adverse effects on short-term outcomes. Caution should be exercised when using opioids during TH until longer-term neurodevelopmental outcome studies can be conducted in larger cohorts.

## 1 Introduction

Therapeutic hypothermia (TH) is the recommended treatment for neonates with signs and symptoms of neonatal encephalopathy (NE) occurring after a significant perinatal event, presumed to be caused by hypoxic ischemia. The clinical presentation of NE includes depressed level of consciousness, decreased spontaneous movement, low muscle tone, poor sucking reflex, and seizures. This commonly occurs in the context of a perinatal sentinel event (e.g., cord prolapse, placental abruption causing hypoxic-ischemic encephalopathy) and can be associated with end-organ involvement and long-term neurodevelopmental sequela. NE is traditionally categorized as mild, moderate, and severe using modified Sarnat scoring and other assessment tools, with TH considered standard treatment for moderate and severe NE (1, 2). Published data demonstrate the benefits of initiating TH in neonates prior to 6 h of life and continuing for 72 h (2–4).

In many centers around the country, agitation, discomfort, and pain during TH are routinely treated with opioids such as morphine and fentanyl (continuous infusion and/or intermittent bolus administration) (1). Morphine has anti-nociceptive properties and reduces the release of glutamate and the sensitivity of glutamate receptors. Glutamate is an excitatory neurotransmitter and is responsible for triggering apoptosis during secondary energy failure characteristic of neonatal NE. This is contrary to what would be expected since morphine inhibits neighboring gamma-aminobutyric acid (GABA) inhibitory neurons, which leads to excitation of ventral tegmental area dopamine (VTA-DA) neurons (5). Secondary energy failure typically occurs approximately 6–15 h after the injury and is characterized by cytotoxic edema, excitotoxicity, and eventually complete failure of mitochondria. Glutamate release activates glutamate and N-methyl-D-aspartate (NMDA) receptors which results in an influx of sodium, chloride and calcium which results in apoptosis and necrosis (6). Inhibition of glutamate release and reduction of glutamate receptor sensitivity is thought to result in neuroprotection (7). Angeles et al. (7) found that neonates who were given morphine during TH had less evidence of hypoxic-ischemic injury on magnetic resonance imaging compared to those who did not receive opioids.

Repeated neonatal pain may be associated with adverse neurodevelopmental outcomes. Williams and Lascelles (8) suggested that long-term consequences of untreated neonatal pain are associated with adverse sensorimotor/cognitive development and subsequent future responses to pain. Walker (9) also reviewed the long-term effects of neonatal pain on neurodevelopment and suggested that there are distinct differences when morphine is administered as a sedative in the absence of pain compared to when used as an analgesic for true pain. Gunderson et al. (10) reported a prospectively collected cohort of 282 neonates with NE treated with TH and exposed to opioids and found no adverse effects on neurodevelopmental outcomes at 18–24 months of age.

Natarajan et al. (11) demonstrated that sedation during TH does not impact neurologic outcomes but suggested that further studies should be studied in neonates exposed to TH. Liow et al. (12) performed a secondary analysis of a large multinational prospective observational study examining the association between morphine infusion during TH on brain injury and neurodevelopmental outcomes. They concluded that morphine does not offer any neuroprotective benefits and may be associated with a prolonged hospital stay (12). Additionally, Frymoyer et al. (13) conducted a pharmacokinetic study in 20 neonates with NE receiving morphine during TH and found clearance to be significantly slower compared to term controls. Similarly, another study found that serum morphine concentrations during the 24–72 h after birth were higher in neonates who underwent TH compared to their normothermic controls despite similar morphine infusion rates and doses (14). The potential adverse effects of opioids (e.g., hypotension, respiratory depression) are especially concerning given that clearance is decreased during TH (13, 14).

The goal of the present study was to determine whether opioid use during TH for moderate and severe NE affects short-term clinical outcomes including length of hospitalization, duration and mode of respiratory support, and need for vasopressor medications for blood pressure support. Our hypothesis was that morphine or fentanyl administration during TH for NE does impact these short-term clinical outcomes. Current research is lacking data regarding these specific short-term clinical outcomes. The goal of our study was to provide further evidence for the potential risks of routine opioid use during TH.

## 2 Methods

### 2.1 Study participants

This is a multicenter retrospective study of neonates receiving TH for moderate or severe NE at Tufts Medical Center (TMC) in Boston MA, Norton Children's Hospital (NCH) in Louisville KY, and Brenner Children's Hospital at Wake Forest (WF) in Winston Salem NC after IRB approval. At all three centers, the severity of NE was determined using Sarnat scoring and neonates with mild NE were excluded even if they received TH (15). Opioid use for sedation during TH is not routine at one center but is standard of care at the other two centers (morphine continuous infusion or (fentanyl continuous infusion). Fentanyl is 100 times more potent than morphine; a dose of only 100 mg (0.1 mg) can produce equivalent analgesia to approximately 10 mg of morphine (16).

### 2.2 Study design

Charts were examined from neonates undergoing TH for moderate or severe NE from January 2013–February 2021. Demographic information related to the prenatal, intrapartum, and postpartum care was collected including pregnancy complications, delivery information, need for neonatal resuscitation, and data regarding the diagnosis of NE and eligibility for TH. Laboratory and imaging data (e.g., liver and kidney function tests, hematologic assessments, coagulation studies, and ultrasound and MRI findings) were used as surrogate assessments for severity of illness. De-identified data from each center were entered into a REDCap database. Due to the retrospective nature of this study, not all data were available for all patients in the study. During the study period, there were no major changes in the clinical approach to neonates receiving TH.

### 2.3 EEG monitoring

The TH protocols at all three centers includes EEG monitoring for accurate identification and treatment of neonatal seizures. The timing for initiation of EEG varied based on the local resources with many neonates from all three centers empirically treated for seizure activity prior to the initiation of continuous video EEG monitoring. In some cases, clinical signs of suspected seizures were not confirmed by video EEG and the use of empiric treatment (that was ultimately stopped) prevented us from using antiepileptic treatment as a marker for seizure activity. Thus, data were evaluated based on EEG proven seizures in neonates who were subsequently treated with antiepileptic medications.

### 2.4 Primary outcome measures in relation to opioid use

The primary outcomes examined in this study were: (1) length of hospitalization, (2) duration and type of respiratory support, and (3) use of vasopressor medications for blood pressure support. Secondary outcomes included time to full oral feeds, duration of parenteral nutrition, and total time a central line was in place. The major focus of this study was exposure to opioids (e.g., morphine, fentanyl) which was classified as positive or negative based on cumulative morphine milligram equivalents (MME). Positive opioid exposure was defined as >0.1 mg/kg cumulative MME and negative as no opioid doses or a very low total of ≤0.1 mg/kg during TH.

### 2.5 Statistical analyses

The clinical and laboratory characteristics of the two opioid exposure groups were analyzed using Wilcoxon rank sum tests for continuous variables and chi-square tests for categorical variables. The clinical and laboratory characteristics of each center were analyzed using Kruskal-Wallis tests for continuous variables and chi-square tests for categorical variables. We used negative binomial regression models to evaluate the association between opioid exposure and length of hospitalization, length of vasopressor use, duration of noninvasive ventilation, time to come off parenteral nutrition, total time a central line was in place, and time to full oral feeds. Linear regression was used to evaluate the association between opioid exposure and log-transformed duration of invasive ventilation. We used logistic regression to evaluate the association between opioid exposure and vasopressor use. All models were adjusted for center and for EEG confirmed seizures (as a marker of disease severity). A two-sided 0.05 level of significance was used. All analyses were conducted using SAS Enterprise Guide software, Version 8.3 Update 3 (SAS Institute Inc., Cary, NC).

## 3 Results

### 3.1 Demographic and perinatal characteristics

We included 120 neonates (males and females) in the study: 52 at Center 1, 39 at Center 2, and 29 at Center 3. Table 1A, B show the maternal and neonatal demographic and perinatal characteristics for neonates in the study, by center and by cumulative MME category or opiate exposure (positive or negative). There were no statistically significant differences between the sites with respect to sex, gestational age, birth weight, and mode of delivery. Most neonates receiving TH at all three centers were outborn (17).

**Table 1A:** Maternal and neonatal demographic and perinatal characteristics analyzed by center.

Variable	All	Center 1	Center 2	Center 3	*p*-value
	(*N* = 120)	(*N* = 52)	(*N* = 39)	(*N* = 29)	
Severe encephalopathy, *n* (%)	14 (11.7)	5 (9.6)	0 (0.0)	9 (31.0)	**0**.**0003**
Inborn (yes), *n* (%)	10 (8.3)	4 (7.7)	2 (5.1)	4 (13.8)	0.40
Male sex, *n* (%)	75 (63.0) [*N* = 119]	32 (61.5)	25 (64.1)	18 (64.3) [*N* = 28]	0.96
Birth weight (g), mean (SD)	3,349.6 (616.1)	3,371.9 (607.7)	3,408.9 (506.2)	3,229.8 (756.5)	0.47
Gestational age (weeks), mean (SD)	38.7 (1.6)	39.0 (1.4)	39.0 (1.4)	37.8 (1.8)	**0**.**003**
Mode of delivery, *n* (%)					
C-Section	75 (62.5)	31 (59.6)	24 (61.5)	20 (69.0)	0.16
Spontaneous vaginal delivery	33 (27.5)	12 (23.1)	14 (35.9)	7 (24.1)	
Vacuum-assisted vaginal delivery	12 (10.0)	9 (17.3)	1 (2.6)	2 (6.9)	
Maternal indication for delivery, *n* (%)					
Abruption	18 (15.0)	5 (9.6)	8 (20.5)	5 (17.2)	0.51
Chorioamnionitis	10 (8.3)	6 (11.5)	4 (10.3)	0 (0.0)	
Chronic hypertension	4 (3.3)	3 (5.8)	0 (0.0)	1 (3.5)	
Gestational diabetes	3 (2.5)	2 (3.9)	0 (0.0)	1 (3.5)	
Maternal fever	1 (0.8)	1 (1.9)	0 (0.0)	0 (0.0)	
Fetal indications for delivery, *n* (%)					
NFHRT/fetal decelerations, *n* (%)	56 (46.7)	30 (57.7)	12 (30.8)	14 (48.3)	**0**.**04**
Failure to progress, *n* (%)	7 (5.8)	2 (3.9)	2 (5.1)	3 (10.3)	0.54
Post-dates, *n* (%)	7 (5.8)	6 (11.5)	1 (2.6)	0 (0.0)	0.07
Cord prolapse, *n* (%)	7 (5.8)	3 (5.8)	3 (7.7)	1 (3.5)	0.89
Delivery data					
Apgar 1 min, median (IQR)	1 (1–2)	1 (1–2)	1 (1–2)	1 (0–2)	0.45
Apgar 5 min, median (IQR)	3 (2–5)	3 (2–4)	4 (2–6)	3 (1–4)	0.03
Apgar 10 min, median (IQR)	5 (3–7) [*N* = 109]	5 (4–6) [*N* = 50]	6 (3–7) [*N* = 35]	5 (2–7) [*N* = 24]	0.28
Cord arterial blood pH, mean (SD)	7.0 (0.2) [*N* = 59]	7.0 (0.2) [*N* = 29]	6.9 (0.2) [*N* = 17]	7.0 (0.1) [*N* = 13]	
Cord arterial BE, mean (SD)	−15.3 (5.5) [*N* = 48]	−14.4 (5.6) [*N* = 21]	−15.9 (5.3) [*N* = 18]	−16.4 (6.0) [*N* = 9]	
Cord venous blood pH, mean (SD)	7.0 (0.2) [*N* = 59]	7.0 (0.2) [*N* = 32]	7.0 (0.2) [*N* = 18]	7.0 (0.2) [*N* = 9]	
Cord venous BE, mean (SD)	−14.5 (6.5) [*N* = 41]	−13.3 (4.2) [*N* = 15]	−15.0 (7.2) [*N* = 17]	−15.7 (8.6) [*N* = 9]	
First neonatal gas pH, mean (SD)	7.1 (0.2) [*N* = 108]	7.1 (0.2) [*N* = 44]	7.1 (0.2) [*N* = 36]	7.1 (0.2) [*N* = 28]	0.70
First neonatal gas BE, mean (SD)	−15.1 (6.3) [*N* = 77]	−15.7 (6.6) [*N* = 19]	−14.5 (6.0) [*N* = 33]	−15.5 (6.5) [*N* = 25]	
Delivery complications, *n* (%)					
Meconium, *n* (%)	28 (23.3)	7 (13.5)	14 (35.9)	7 (24.1)	**0**.**04**
Shoulder dystocia, *n* (%)	15 (12.5)	7 (13.5)	6 (15.4)	2 (6.9)	0.61
Nuchal cord, *n* (%)	15 (12.5)	7 (13.5)	5 (12.8)	3 (10.3)	1.00
Breech/footling breech, *n* (%)	5 (4.2)	0 (0.0)	1 (2.6)	4 (13.8)	
Neonatal resuscitation at delivery, *n* (%)					
CPAP, *n* (%)	18 (15.0)	13 (25.0)	0 (0.0)	5 (17.2)	**0**.**004**
PPV with bag/mask/O2, *n* (%)	113 (94.2)	47 (90.4)	39 (100.0)	27 (93.1)	0.14
Intubation, *n* (%)	87 (72.5)	35 (67.3)	31 (79.5)	21 (72.4)	0.44
Normal saline, *n* (%)	23 (19.2)	5 (9.6)	9 (23.1)	9 (31.0)	**0**.**04**
Epinephrine, *n* (%)	22 (18.3)	7 (13.5)	5 (12.8)	10 (34.5)	0.05

NRFHT, non-reassuring fetal heart tracing; BE, base excess; PPV, positive pressure ventilation.

Bold value for *p* value represents statistical significance at *p* value <0.05.

**Table 1B:** Maternal and neonatal demographics and perinatal characteristics analyzed by cumulative MME and opioid exposure categories.

Variable	All (*N* = 120)	Positive exposure (>0.1 mg/kg) (*N* = 82)	Negative exposure (≤0.1 mg/kg) (*N* = 38)	*p*-value
Severe encephalopathy, *n* (%)	14 (11.7)	8 (9.8)	6 (15.8)	0.37
Inborn (yes), *n* (%)	10 (8.3)	5 (6.1)	5 (13.2)	0.29
Male sex, *n* (%)	75 (63.0) [*N* = 119]	52 (63.4)	23 (62.2) [*N* = 37]	0.90
Hospital, *n* (%)				
Center 1	52 (43.3)	23 (28.1)	29 (76.3)	**<0**.**0001**
Center 2	39 (32.5)	38 (46.3)	1 (2.6)	
Center 3	29 (24.2)	21 (25.6)	8 (21.1)	
Birth weight (g), mean (SD)	3,349.6 (616.1)	3,449.7 (584.8)	3,133.6 (634.1)	**0**.**01**
Gestational age (weeks), mean (SD)	38.7 (1.6)	38.8 (1.4)	38.4 (1.8)	0.20
Mode of delivery, *n* (%)				
C-Section	75 (62.5)	51 (62.2)	24 (63.2)	0.65
Spontaneous vaginal delivery	33 (27.5)	24 (29.3)	9 (23.7)	
Vacuum-assisted vaginal delivery	12 (10.0)	7 (8.5)	5 (13.2)	
Maternal indication for delivery, *n* (%)				
Abruption	18 (15.0)	13 (15.9)	5 (13.2)	0.48
Chorioamnionitis	10 (8.3)	5 (6.1)	5 (13.2)	
Chronic hypertension	4 (3.3)	2 (2.4)	2 (5.3)	
Gestational diabetes	3 (2.5)	3 (3.7)	0 (0.0)	
Maternal fever	1 (0.8)	0 (0.0)	1 (2.6)	
Pre-eclampsia	4 (3.3)	2 (2.4)	2 (5.3)	
Fetal indications for delivery, *n* (%)				
NFHRT/fetal decelerations, *n* (%)	56 (46.7)	35 (42.7)	21 (55.3)	0.20
Failure to progress, *n* (%)	7 (5.8)	5 (6.1)	2 (5.3)	
Post-dates, *n* (%)	7 (5.8)	6 (7.3)	1 (2.6)	
Cord prolapse, *n* (%)	7 (5.8)	5 (6.1)	2 (5.3)	
Delivery information				
Apgar 1 min, median (IQR)	1 (1–2)	1 (0–2)	1 (1–2)	0.23
Apgar 5 min, median (IQR)	3 (2–5)	3 (1–5)	3 (2–4)	0.84
Apgar 10 min, median (IQR)	5 (3–7) [*N* = 109]	5 (3–7) [*N* = 73]	5 (3–7) [*N* = 36]	0.69
Cord arterial blood pH, mean (SD)	7.0 (0.2) [*N* = 59]	7.0 (0.2) [*N* = 35]	6.9 (0.2) [*N* = 24]	
Cord arterial BE, mean (SD)	−15.3 (5.5) [*N* = 48]	−14.8 (5.1) [*N* = 31]	−16.2 (6.3) [*N* = 17]	
Cord venous blood pH, mean (SD)	7.0 (0.2) [*N* = 59]	7.0 (0.2) [*N* = 38]	7.0 (0.1) [*N* = 21]	
Cord venous BE, mean (SD)	−14.5 (6.5) [*N* = 41]	−13.3 (6.6) [*N* = 30]	−17.9 (5.2) [*N* = 11]	
First neonatal gas obtained pH, mean (SD)	7.1 (0.2) [*N* = 108]	7.1 (0.2) [*N* = 73]	7.1 (0.2) [*N* = 35]	0.16
First neonatal gas BE, mean (SD)	−15.1 (6.3) [*N* = 77]	−15.0 (6.6) [*N* = 58]	−15.4 (5.3) [*N* = 19]	
Delivery complications, *n* (%)				
Meconium, *n* (%)	28 (23.3)	23 (28.1)	5 (13.2)	0.07
Shoulder dystocia, *n* (%)	15 (12.5)	13 (15.9)	2 (5.3)	0.14
Nuchal cord, *n* (%)	15 (12.5)	10 (12.2)	5 (13.2)	1.00
Neonatal resuscitation at delivery, *n* (%)				
CPAP, *n* (%)	18 (15.0)	10 (12.2)	8 (21.1)	0.21
PPV with bag/mask/O2, *n* (%)	113 (94.2)	79 (96.3)	34 (89.5)	0.21
Intubation, *n* (%)	87 (72.5)	60 (73.2)	27 (71.1)	0.81
Normal saline, *n* (%)	23 (19.2)	18 (22.0)	5 (13.2)	0.26
Epinephrine, *n* (%)	22 (18.3)	19 (23.2)	3 (7.9)	**0**.**04**

NRFHT, non-reassuring fetal heart tracing; BE, base excess; PPV, positive pressure ventilation.

Bold value for *p* value represents statistical significance at *p* value <0.05.

### 3.2 Clinical findings

Severity of NE varied among the centers, with 31% classified as severe at one center (Table 1A). At all three centers, placental abruption was the most common maternal indication for delivery and non-reassuring fetal heart tracing was the most common fetal indication with overlap between all three institutions. Significant differences were noted in the documentation of non-reassuring fetal heart rate tracings. The use of resuscitative measures, delivery complications, and laboratory data were comparable among centers, including the degree of acidosis on the cord and/or first postnatal blood gas (Table 1A). The severity of acidosis was similar between sites during cooling and rewarming periods (first 6 days of the hospital stay).

### 3.3 Primary outcomes

The unadjusted analyses of the primary outcomes are shown in Table 2A, B. Table 2A compares the exposure and the outcomes across centers and Table 2B compares the exposure and outcomes between exposure categories. The cumulative opioid dosing analysis demonstrated that 68% of all neonates in the study were exposed to >0.1 mg/kg MME. Analysis revealed numerous inter-center differences including percent of opioid-exposed neonates, cumulative opioid-equivalent dose, vasopressor use (not the duration of use), duration of mechanical ventilation, central line use, time to full oral feedings, and length of hospital stay. Table 3A, B contain additional clinical and laboratory data analyzed by center as well as by cumulative opioid exposure with no statistically significant differences between the two opioid exposure groups. Tables 4–7 show the analysis of the primary outcomes by center, after adjusting for opioid exposure and EEG confirmed seizure activity.

**Table 2A:** Primary and secondary outcomes analyzed by center.

Variable	All (*N* = 120)	Center 1 (*N* = 52)	Center 2 (*N* = 39)	Center 3 (*N* = 29)	*p*-value
Opioid administered, *n* (%)					
Morphine alone	75 (62.5)	37 (71.2)	38 (97.4)	0 (0.0)	**<0**.**0001**
Fentanyl alone	22 (18.3)	0 (0.0)	0 (0.0)	22 (75.9)	
Morphine and fentanyl	1 (0.8)	0 (0.0)	0 (0.0)	1 (3.5)	
None	22 (18.3)	15 (28.9)	1 (2.6)	6 (20.7)	
Cumulative MME (mg/kg), median (IQR)	0.8 (0.2–1.6) [*N* = 98]	0.2 (0.1–0.4) [*N* = 37]	0.9 (0.8–1.6) [*N* = 38]	3.3 (0.8–8.1) [*N* = 23]	**<0**.**0001**
Cumulative MME categories, *n* (%)					
High (>0.1 mg/kg)	82 (68.3)	23 (44.2)	38 (97.4)	21 (72.4)	**<0**.**0001**
Low (≤0.1 mg/kg)	38 (31.7)	29 (55.8)	1 (2.6)	8 (27.6)	
Outcome variables					
Length of stay (days), median (IQR)	13 (9–23)	9 (8–20)	14 (12–22)	23 (10–34)	**0**.**004**
Vasopressors, *n* (%)	50 (41.7)	12 (23.1)	26 (66.7)	12 (41.4)	**0**.**0002**
Multiple vasopressors, *n* (%)	18 (15.0)	4 (7.7)	7 (18.0)	7 (24.1)	0.11
Duration of vasopressor use (days), median (IQR)	3 (2–5) [*N* = 50]	3 (1–4) [*N* = 12]	4 (3–5) [*N* = 26]	3 (2–6) [*N* = 12]	0.10
Invasive ventilation, *n* (%)	93 (77.5)	35 (67.3)	33 (84.6)	25 (86.2)	0.06
Length of invasive ventilation (days), median (IQR)	2 (1–5) [*N* = 93]	2 (1–3) [*N* = 35]	3 (2–6) [*N* = 33]	5 (1–8) [*N* = 25]	**0**.**01**
Non-invasive ventilation, *n* (%)	87 (72.5)	36 (69.2)	39 (100.0)	12 (41.4)	**<0**.**0001**
Length of non-invasive ventilation (days), median (IQR)	3 (2–4) [*N* = 87]	2 (1–4) [*N* = 36]	3 (2–4)	4 (1–7) [*N* = 12]	0.35
Duration of time off PN prior to discharge (days), median (IQR)	6 (3–13) [*N* = 117]	3 (2–11) [*N* = 51]	7 (4–10) [*N* = 38]	13 (5–19) [*N* = 28]	**0**.**01**
Duration of central line (days), median (IQR)	7 (5–9) [*N* = 119]	6 (5–8)	8 (7–10)	7 (6–10) [*N* = 28]	**0**.**003**
Time to full oral feeds (DOL), median (IQR)	7 (6–12) [*N* = 106]	6 (5–7) [*N* = 45]	9 (7–14)	8 (7–15) [*N* = 22]	**0**.**0002**

PN, parenteral nutrition; DOL, day of life.

Bold value for *p* value represents statistical significance at *p* value <0.05.

**Table 2B:** Primary and secondary outcomes analyzed by cumulative MME and opioid exposure categories.

Variable	All (*N* = 120)	Positive exposure (>0.1 mg/kg) (*N* = 82)	Negative exposure. (≤0.1 mg/kg) (*N* = 38)	*p*-value
Morphine alone	75 (62.5)	61 (74.4)	14 (36.8)	**<0**.**0001**
Fentanyl alone	22 (18.3)	20 (24.4)	2 (5.3)	
Morphine and fentanyl	1 (0.8)	1 (1.2)	0 (0.0)	
None	22 (18.3)	0 (0.0)	22 (57.9)	
Cumulative MME (mg/kg), median (IQR)	0.5 (0.1–1.2)	0.9 (0.4–2.0)	0.0 (0.0–0.1)	
Outcome variables				
Length of stay (days), median (IQR)	13 (9–23)	14 (9–24)	11 (8–21)	0.12
Vasopressors, *n* (%)	50 (41.7)	40 (48.8)	10 (26.3)	**0**.**02**
Multiple vasopressors, *n* (%)	18 (15.0)	13 (15.9)	5 (13.2)	0.70
Duration of vasopressor use (days), median (IQR)	3 (2–5) [*N* = 50]	4 (2–5) [*N* = 40]	3 (1–3) [*N* = 10]	0.08
Invasive ventilation, *n* (%)	93 (77.5)	65 (79.3)	28 (73.7)	0.50
Length of invasive ventilation (days), median (IQR)	2 (1–5) [*N* = 93]	3 (1–7) [*N* = 65]	2 (1–3) [*N* = 28]	**0**.**03**
Noninvasive ventilation, *n* (%)	87 (72.5)	65 (79.3)	22 (57.9)	**0**.**01**
Length of noninvasive ventilation (days), median (IQR)	3 (2–4) [*N* = 87]	3 (2–4) [*N* = 65]	3 (1–7) [*N* = 22]	0.60
Duration of time off PN prior to discharge (days), median (IQR)	6 (3–13) [*N* = 117]	7 (3–13) [*N* = 79]	4 (2–11)	0.11
Duration of central line (days), median (IQR)	7 (5–9) [*N* = 119]	7 (6–10)	6 (5–7) [*N* = 37]	**0**.**01**
Time to full oral feeds (DOL), median (IQR)	7 (6–12) [*N* = 106]	8 (6–13) [*N* = 73]	6 (5–7) [*N* = 33]	**0**.**01**

PN, parenteral nutrition; DOL, day of life; MME, morphine mEq.

Bold value for *p* value represents statistical significance at *p* value <0.05.

**Table 3A:** Clinical and laboratory findings analyzed by center.

Variable	All (*N* = 120)	Center 1 (*N* = 52)	Center 2 (*N* = 39)	Center 3 (*N* = 29)	*p*-value
Clinical findings					
Sepsis, *n* (%)	5 (4.2)	1 (1.9)	3 (7.7)	1 (3.5)	0.44
Pneumothorax, *n* (%)	8 (6.7)	3 (5.8)	3 (7.7)	2 (6.9)	1.00
Intraventricular hemorrhage, *n* (%)	9 (7.6) [*N* = 119]	1 (1.9)	3 (7.7)	5 (17.9) [*N* = 28]	**0**.**03**
Nitric oxide use, *n* (%)	18 (15.0)	4 (7.7)	6 (15.4)	8 (27.6)	0.05
Steroids use, *n* (%)	25 (20.8)	2 (3.9)	13 (33.3)	10 (34.5)	**0**.**0003**
Initiation of trophic feeds (day of life), median (IQR)	4 (4–5) [*N* = 118]	4 (4–4)	5 (4–7)	4 (3–6) [*N* = 27]	**0**.**001**
Gastrostomy tube at discharge, *n* (%)	11 (9.2)	5 (9.6)	0 (0.0)	6 (20.7)	**0**.**01**
Laboratory values					
AST (highest), median (IQR)	161 (101–326) [*N* = 119]	150 (88–383) [*N* = 51]	191 (109–326)	161 (81–288)	0.76
ALT (highest), median (IQR)	66 (29–163) [*N* = 119]	64 (28–160) [*N* = 51]	96 (41–239)	44 (18–127)	**0**.**02**
BUN (highest), median (IQR)	20 (14–27)	23 (19–29)	16 (11–20)	22 (14–27)	**<0**.**0001**
Creatinine (highest), median (IQR)	0.9 (0.8–1.2)	1.0 (0.8–1.4)	0.8 (0.7–0.9)	1.0 (0.8–1.3)	**0**.**002**
Bicarbonate (lowest), median (IQR)	15 (12–18) [*N* = 102]	15 (12–18) [*N* = 51]	16 (13–20) [*N* = 22]	14 (11–17)	0.30
Partial thromboplastin time (PTT) (highest), median (IQR)	59.7 (43.5–80.9) [*N* = 95]	60.6 (48.5–93.4) [*N* = 47]	62.4 (45.2–77.4)	39.6 (30.0–71.3) [*N* = 9]	0.06
INR, median (IQR)	1.5 (1.4–2.0) [*N* = 97]	1.5 (1.4–1.9) [*N* = 47]	1.6 (1.3–2.1)	1.7 (1.4–2.6) [*N* = 11]	0.44
Fibrinogen (lowest), median (IQR)	154 (116–191) [*N* = 95]	152 (103–172) [*N* = 46]	159 (133–225)	134 (105–176) [*N* = 10]	0.26
Hematocrit, median (IQR)	42.0 (34.5–47.2)	43.3 (35.8–47.5)	39.3 (32.9–47.0)	40.1 (32.8–47.0)	0.44
Platelets (lowest), median (IQR)	133 (71–182) [*N* = 119]	147 (92–202) [*N* = 51]	102 (62–161)	139 (69–195)	**0**.**04**
Seizure classification					
No seizures, *n* (%)	66 (55.0)	33 (63.5)	21 (53.9)	12 (41.4)	0.15
Clinical seizures, *n* (%)	32 (26.7)	10 (19.2)	14 (35.9)	8 (27.6)	0.20
EEG-confirmed, *n* (%)	24 (20.0)	9 (17.3)	4 (10.3)	11 (37.9)	**0**.**02**
Anti-seizure medications, *n* (%)	59 (49.2)	20 (38.5)	18 (46.2)	21 (72.4)	**0**.**01**
Head ultrasound findings, *n* (%)					
Normal	69 (57.5)	28 (53.9)	33 (84.6)	8 (27.6)	**<0**.**0001**
Abnormal	25 (20.8)	8 (15.4)	6 (15.4)	11 (37.9)	
Not done	26 (21.7)	16 (30.8)	0 (0.0)	10 (34.5)	
MRI #1, *n* (%)					
Normal	38 (31.7)	12 (23.1)	24 (61.5)	2 (6.9)	**<0**.**0001**
No signs of hypoxic injury	34 (28.3)	22 (42.3)	7 (18.0)	5 (17.2)	
Mild hypoxic ischemic injury	16 (13.3)	4 (7.7)	4 (10.3)	8 (27.6)	
Profound hypoxic ischemic injury	27 (22.5)	14 (26.9)	4 (10.3)	9 (31.0)	
Not done	4 (3.3)	0 (0.0)	0 (0.0)	4 (13.8)	
Unknown	1 (0.8)	0 (0.0)	0 (0.0)	1 (3.5)	
MRI #2, *n* (%)					
Normal	10 (8.3)	6 (11.5)	1 (2.6)	3 (10.3)	**<0**.**0001**
No signs of hypoxic injury	14 (11.7)	14 (26.9)	0 (0.0)	0 (0.0)	
Mild hypoxic ischemic injury	1 (0.8)	1 (1.9)	0 (0.0)	0 (0.0)	
Profound hypoxic ischemic injury	8 (6.7)	7 (13.5)	0 (0.0)	1 (3.5)	
Not done	87 (72.5)	24 (46.2)	38 (97.4)	25 (86.2)	

AST, aspartate transaminase; ALT, alanine transaminase; EEG, electroencephalogram.

Bold value for *p* value represents statistical significance at *p* value <0.05.

**Table 3B:** Clinical and laboratory findings analyzed by cumulative MME and opioid exposure categories.

Variable	All (*N* = 120)	Positive exposure (>0.1 mg/kg) (*N* = 82)	Negative exposure (≤0.1 mg/kg) (*N* = 38)	*p*-value
Clinical findings				
Pneumothorax, *n* (%)	8 (6.7)	6 (7.3)	2 (5.3)	1.00
Intraventricular hemorrhage, *n* (%)	9 (7.6) [*N* = 119]	8 (9.9) [*N* = 81]	1 (2.6)	0.27
Nitric oxide use, *n* (%)	18 (15.0)	14 (17.1)	4 (10.5)	0.35
Steroid use, *n* (%)	25 (20.8)	22 (26.8)	3 (7.9)	**0**.**02**
Initiation of trophic feeds (day of life), median (IQR)	4 (4–5) [*N* = 118]	4 (4–5) [*N* = 80]	4 (3–5)	**0**.**01**
Gastrostomy tube at discharge, *n* (%)	11 (9.2)	7 (8.5)	4 (10.5)	0.74
Laboratory values				
AST (highest), median (IQR)	161 (101–326) [*N* = 119]	150 (95–326) [*N* = 81]	210 (105–298)	0.45
ALT (highest), median (IQR)	66 (29–163) [*N* = 119]	57 (31–160) [*N* = 81]	83 (28–163)	0.97
BUN (highest), median (IQR)	20 (14–27)	20 (13–26)	21 (17–29)	0.09
Creatinine (highest), median (IQR)	0.9 (0.8–1.2)	0.8 (0.7–1.1)	1.1 (0.8–1.6)	**0**.**0006**
Bicarbonate (lowest), median (IQR)	15 (12–18) [*N* = 102]	15 (12–19) [*N* = 64]	14 (12–17)	**0**.**03**
Partial thromboplastin time (PTT) (highest), median (IQR)	59.7 (43.5–80.9) [*N* = 95]	54.3 (42.1–73.7) [*N* = 65]	71.4 (44.9–116.1) [*N* = 30]	0.06
INR, median (IQR)	1.5 (1.4–2.0) [*N* = 97]	1.5 (1.4–2.1) [*N* = 66]	1.5 (1.4–1.9) [*N* = 31]	0.68
Fibrinogen (lowest), median (IQR)	154 (116–191) [*N* = 95]	157 (117–209) [*N* = 64]	151 (105–172) [*N* = 31]	0.31
Hematocrit, median (IQR)	42.0 (34.5–47.2)	41.2 (34.5–46.6)	43.0 (34.4–47.8)	0.87
Platelets (lowest), median (IQR)	133 (71–182) [*N* = 119]	114 (70–171)	147 (93–200) [*N* = 37]	0.11
Seizure classification, *n* (%)				
No seizures, *n* (%)	66 (55.0)	45 (54.9)	21 (55.3)	0.97
Clinical seizures, *n* (%)	32 (26.7)	24 (29.3)	8 (21.1)	0.34
EEG-confirmed, *n* (%)	24 (20.0)	14 (17.1)	10 (26.3)	0.24
Anti-seizure medications, *n* (%)	59 (49.2)	39 (47.6)	20 (52.6)	0.61
Head ultrasound findings, *n* (%)				
Normal	69 (57.5)	49 (59.8)	20 (52.6)	0.42
Abnormal	25 (20.8)	18 (22.0)	7 (18.4)	
Not done	26 (21.7)	15 (18.3)	11 (29.0)	
MRI #1, *n* (%)				
Normal	38 (31.7)	33 (40.2)	5 (13.2)	**0**.**02**
No signs of hypoxic injury	34 (28.3)	20 (24.4)	14 (36.8)	
Mild hypoxic ischemic injury	16 (13.3)	12 (14.6)	4 (10.5)	
Profound hypoxic ischemic injury	27 (22.5)	13 (15.9)	14 (36.8)	
Not done	4 (3.3)	3 (3.7)	1 (2.6)	
Unknown	1 (0.8)	1 (1.2)	0 (0.0)	
MRI #2, *n* (%)				
Normal	10 (8.3)	4 (4.9)	6 (15.8)	**0**.**01**
No signs of hypoxic injury	14 (11.7)	8 (9.8)	6 (15.8)	
Mild hypoxic ischemic injury	1 (0.8)	0 (0.0)	1 (2.6)	
Profound hypoxic ischemic injury	8 (6.7)	3 (3.7)	5 (13.2)	
Not done	87 (72.5)	67 (81.7)	20 (52.6)	

AST, aspartate transaminase; ALT, alanine transaminase; EEG, electroencephalogram.

Bold value for *p* value represents statistical significance at *p* value <0.05.

**Table 4:** Inter-center comparison of length of stay analyzed by opioid exposure categories (*N* = 120).

Adjusting for center				
Variable	Exponentiated estimate	95% confidence limits		*p*-value
High vs. low cumulative opioid dose	0.97	0.75	1.27	0.8338
Center 2 vs. Center 1	1.22	0.91	1.62	0.1796
Center 3 vs. Center 1	1.62	1.23	2.15	0.0006
Center 3 vs. Center 2	1.34	1.23	1.46	0.0438
Adjusting for center and EEG-confirmed seizure				
Variable	Exponentiated estimate	95% confidence limits		*p*-value
High vs. low cumulative opioid dose	1.05	0.82	1.34	0.6978
Center 2 vs. Center 1	1.27	0.98	1.65	0.0763
Center 3 vs. Center 1	1.49	1.15	1.92	0.0025
Center 3 vs. Center 2	1.17	0.9	1.54	0.2467
EEG-confirmed seizure	1.72	1.35	2.2	<.0001

**Table 5:** Inter-center comparison of vasopressor use analyzed by opioid exposure categories (*N* = 120).

Adjusting for center				
Variable	Odds ratio	95% confidence limits		*p*-value
High vs. low cumulative opioid dose	1.19	0.44	3.2	0.735
Center 2 vs. Center 1	6.1	2.12	17.52	0.001
Center 3 vs. Center 1	2.25	0.81	6.2	0.119
Center 3 vs. Center 2	0.37	0.13	1.03	0.056
Adjusting for center and EEG-confirmed seizure				
Variable	Odds ratio	95% confidence limits		*p*-value
High vs. low cumulative opioid dose	1.31	0.47	3.65	0.605
Center 2 vs. Center 1	6.53	2.21	19.26	0.001
Center 3 vs. Center 1	1.8	0.62	5.25	0.282
Center 3 vs. Center 2	0.28	0.09	0.82	0.021
EEG-confirmed seizure	2.72	0.98	7.58	0.056

**Table 6:** Inter-center comparison of duration of invasive ventilation analyzed by opioid exposure categories (*N* = 93).

Adjusting for center				
Variable	Exponentiated estimate	95% confidence limits		*p*-value
High vs. low cumulative opioid dose	1.26	0.87	1.83	0.214
Center 2 vs. Center 1	1.26	0.85	1.88	0.247
Center 3 vs. Center 1	1.63	1.11	2.4	0.014
Center 3 vs. Center 2	1.29	0.89	1.88	0.181
Adjusting for center and EEG-confirmed seizure				
Variable	Exponentiated Estimate	95% Confidence Limits		*p*-value
High vs. low cumulative opioid dose	1.32	0.92	1.90	0.127
Center 2 vs. Center 1	1.29	0.88	1.90	0.1915
Center 3 vs. Center 1	1.46	0.99	2.14	0.0545
Center 3 vs. Center 2	1.13	0.77	1.65	0.524
EEG-confirmed seizure	1.57	1.12	2.20	0.010

**Table 7:** Inter-center comparison of length of noninvasive ventilation analyzed by opioid exposure categories (*N* = 87).

Adjusting for center				
Variable	Exponentiated estimate	95% confidence limits		*p*-value
High vs. low cumulative opioid dose	0.60	0.38	0.94	0.027
Center 2 vs. Center 1	1.35	0.87	2.1	0.177
Center 3 vs. Center 1	1.24	0.72	2.13	0.437
Center 3 vs. Center 2	0.91	0.54	1.55	0.74
Adjusting for center and EEG-confirmed seizure				
Variable	Exponentiated estimate	95% confidence limits		*p*-value
High vs. low cumulative opioid dose	0.68	0.45	1.04	0.077
Center 2 vs. Center 1	1.41	0.94	2.11	0.1
Center 3 vs. Center 1	1.07	0.65	1.77	0.784
Center 3 vs. Center 2	0.76	0.46	1.25	0.285
EEG-confirmed seizure	2.29	1.54	3.4	<.0001

### 3.4 EEG confirmed seizures

When evaluating exposure to anti-epileptic medications, 35.9% (14 out of 39) of neonates in the positive opioid exposure group had EEG confirmed seizures compared to 50% (10 out of 20) in the negative exposure group. By center, out of the subjects who received anti-epileptic medications, EEG confirmed seizures occurred in 22.2% (4 out of 18) at Center 1, 45% (9 out of 20) at Center 2, and 52.4% (11 out of 21) at Center 3.

### 3.5 Inter-center differences

Inter-center differences persisted after adjusting for center, EEG confirmed seizures, and opioid exposure. Neonates at the center using routine fentanyl had 1.49 times longer length of stay relative to the center with limited opioid use (ratio of means: 1.49, 95% CI: 1.15–1.92; *p* = 0.0025). Neonates with EEG-confirmed seizures had an average length of stay 1.72 times longer as those without seizures (95% CI: 1.35–2.20; *p* < .0001) and were 2.72 times more likely to receive vasopressors (95% CI: 0.98–7.58; *p* = 0.056). Neonates at the center using routine morphine had 6.53 times the odds of vasopressor use compared to the center with limited opioid use (95% CI: 2.21–19.26; *p* = 0.001). Neonates at the center using fentanyl had 0.28 times the odds of vasopressor use compared to the center using morphine (95% CI: 0.09–0.82; *p* = 0.021).

### 3.6 EEG-confirmed seizures and primary outcomes

Neonates with EEG-confirmed seizures had an average length of invasive ventilation 1.57 times longer (95% CI: 1.12–2.20; *p* = 0.01) and non-invasive ventilation 2.29 times longer than those without seizures (95% CI: 1.54–3.40; *p* < .0001).

## 4 Discussion

The benefits and risks of opioid use during TH remain controversial. While some pre-clinical models suggest that morphine may enhance the neuroprotective effects of TH by reducing oxidative stress, other animal studies have suggested that morphine induces neuronal apoptosis which could be harmful (4, 18, 19). The aim of this study was to examine short-term outcomes in neonates requiring TH and exposed to no or minimal doses of opioids compared to those receiving higher MME at three academic medical centers. The results suggest that higher cumulative doses of MME during TH for NE (often via continuous infusions) are associated with a greater need and longer duration of mechanical ventilatory support, vasopressors to support blood pressure, and a longer hospital stay. This is likely due to the direct effects of opioids on respiratory control centers of the brain and on vasomotor tone of blood vessels; effects that may be accentuated when combined with TH (which may delay opioid clearance). The TH protocols at the three centers are similar except for the approach to sedation. Intermittent morphine doses (0.05–0.1 mg/kg) are used only as needed at Center 1, routine administration of morphine by continuous infusion occurs at Center 2, and routine administration of fentanyl by continuous infusion occurs at Center 3. Our analysis demonstrated that the cumulative MME dose was significantly different among the three sites which was somewhat unexpected since the general perception is that continuous infusion decreases the cumulative MME amount required for pain control and comfort.

Our analysis also demonstrated that the cumulative MME dose was significantly different among the three sites with the centers using continuous infusions having the highest cumulative MME dose exposure. This was somewhat unexpected since the general perception is that continuous infusion decreases the cumulative MME amount required for control of pain and discomfort. The substantial difference in the cumulative MME dose at one center compared to the other two centers is related to the preferential use of fentanyl, with significantly higher analgesic potency (100 times) compared to morphine.

Although patient demographics at the three centers were similar, some differences were identified. Most neonates receiving TH at all three centers were outborn which is consistent with current evidence (17). There was a higher number of mothers with substance use disorder in the Center 2 cohort and anxiety and depression at Center 1 (20, 21). The data also showed minor differences in practice patterns among sites with respect to modes of ventilation, fluid resuscitation, laboratory data, and initiation of feeds (Table 3A). Neonates with EEG confirmed seizures and subsequent exposure to antiepileptic medications in addition to opioids required a longer hospitalization, a greater need for vasopressors, and greater need and duration of both invasive and non-invasive ventilation. Duration of vasopressor use though was not found to be statistically significant.

This is a pragmatic study, which enabled the comparison of effect of opioids on short-term outcomes at three centers with similar TH management, despite some differences in clinical practice (e.g., opioid administration). The difference in pressor use could be partially related to variations in the approach to blood pressure measurement at the three institutions. Variations in the use of intra-arterial lines for blood pressure monitoring may influence vasopressor use and contribute to the duration of central line use (22). Higher use of vasopressors may also result in slower advancement of feeds and may partially explain why the delay in initiation trophic feeds. Likewise, differences in ventilation may also be due to the individual center practice or provider preferences regarding the timing of extubation and use of non-invasive ventilation.

The main limitations of our study include the relatively small sample size, retrospective data collection, and clinical practice variations between the three institutions despite very similar TH protocols. In addition, we did not analyze standardized pain scores (due to their subjective nature) among sites, which could potentially have influenced opioid use. Since the concomitant use of anti-seizure medication may be a confounding variable influencing our primary and secondary outcomes, EEG confirmed seizures was used to adjust analyses. The criteria for EEG confirmed seizures was chosen over exposure to antiepileptic medication criteria because many neonates treated empirically with antiepileptic medications did not have EEG proven seizure activity and the medications were stopped (Table 3A, B). At all three centers, only a small percentage of neonates that received anti-epileptics also had EEG confirmed seizures. Another limitation was the lack of an objective tool to more accurately compare the severity of NE and any pain/discomfort associated with TH. Information used as a surrogate biomarker included APGAR scores, blood gases, the presence of multi-organ involvement, and Sarnat scoring. Data collected in this study showed that neonates with a diagnosis of severe NE did not consistently have evidence of multi-organ involvement or the most severe MRI findings.

Although the use of MRI as a biomarker of underlying brain injury is still being debated, MRI findings do correlate with future neurodevelopmental outcomes associated with NE (23). The discrepancy between unfavorable short-term outcomes and MRI results as a long-term prognostic tool emphasizes the need for long-term outcome data to definitively determine benefit or harm of opioid use in the management of neonatal NE during TH. For this study, each center's MRIs were interpreted by their own pediatric neuroradiologist. We were not able to have a single blinded radiologist interpret the results for all the MRIs and this prevented us from formulating statistical conclusions regarding the MRI findings.

The cumulative impact of opioids on long-term neurodevelopmental outcomes after TH could not be analyzed given the limited amounts of follow-up data available from the three centers. Long-term outcomes are critical to establish safety of these medication practices. The optimal therapeutic approach to neonatal pain management using opioids is still unresolved and a standardized approach is lacking. Newer sedatives such as dexmedetomidine are emerging as alternative options and their use in neonates is becoming much more widespread without sufficient compelling data on safety and dosing, especially during TH when the metabolism of many drugs change (24).

This study is one of the few involving real-world data, an important approach considering the paucity of data on the safety and efficacy of opioid use in neonates (especially those with underlying brain injury). While the current results raise concerns regarding the use of opioids in neonates undergoing TH, the findings indicate the complexity of a pragmatic approach to study opioid therapy in TH. A larger multicenter trial with standardized approach to opioid therapy and a detailed pharmacokinetic analyses and comprehensive neurodevelopmental follow-up will ultimately be needed to further investigate the short- and long-term safety and efficacy of opioids during TH.

## Data Availability

The datasets presented in this article are not readily available because N/A. Requests to access the datasets should be directed to tinaj205@gmail.com.
